# Lateral Walking Gait Recognition and Hip Angle Prediction Using a Dual-Task Learning Framework

**DOI:** 10.34133/cbsystems.0250

**Published:** 2025-05-01

**Authors:** Mingxiang Luo, Meng Yin, Jinke Li, Ying Li, Worawarit Kobsiriphat, Hongliu Yu, Tiantian Xu, Xinyu Wu, Wujing Cao

**Affiliations:** ^1^ Guangdong Provincial Key Lab of Robotics and Intelligent System, Shenzhen Institutes of Advanced Technology, Chinese Academy of Sciences, Shenzhen, China.; ^2^ Department of Mechanical and Electrical Engineering, Shenzhen Polytechnic University, Shenzhen, China.; ^3^ National Metal and Materials Technology Center, National Science and Technology Development Agency of Thailand, Khlong Nueng, Thailand.; ^4^ University of Shanghai for Science and Technology, Shanghai, China.

## Abstract

Lateral walking exercise is beneficial for the hip abductor enhancement. Accurate gait recognition and continuous hip joint angle prediction are essential for the control of exoskeletons. We propose a dual-task learning framework, the “Twin Brother” model, which fuses convolutional neural network (CNN), long short-term memory (LSTM), neural networks (NNs), and the squeezing-elicited attention mechanism to classify the lateral gait stage and estimate the hip angle from electromyography (EMG) signals. The EMG signals of 6 muscles from 10 subjects during lateral walking were collected. Four gait phases were recognized, and the hip angles of both legs were continuously estimated. The sliding window length of 250 ms and the sliding increment of 3 ms were determined by the requirements of response time and recognition accuracy of the real-time system. We compared the performance of CNN-LSTM, CNN, LSTM, support vector machine, NN, K-nearest neighbor, and the “Twin Brother” models. The “Twin Brother” model achieved a recognition accuracy (mean ± SD) of 98.81% ± 0.14%. The model’s predicted root mean square error (RMSE) for the left and right hip angles are 0.9183° ± 0.024° and 1.0511° ± 0.027°, respectively, where the *R*^2^ are 0.9853 ± 0.006 and 0.9808 ± 0.008. The accuracy of recognition and estimation are both better than comparative models. For gait phase percentage prediction, RMSE and *R*^2^ predicted by the model can reach 0.152° ± 0.014° and 0.986 ± 0.011, respectively. These results demonstrate that the method can be applied to lateral walking gait recognition and hip joint angle prediction.

## Introduction

In patients undergoing lower limb rehabilitation, weakness in the hip’s adductor and abductor muscles can lead to abnormal gait and increased risk. These muscles stabilize the pelvis and maintain normal gait mechanics [1]. Strengthening them through hip exercises is necessary. Lateral walking benefits the hip abductor muscles substantially [2]. However, effective lateral walking exercises require proper guidance and resistance. Using elastic bands for lateral walking is common but lacks uniform and controlled resistance. More effective solutions are needed. The hip exoskeleton is a promising tool for enhancing muscle activation during lateral walking exercises by providing controlled resistance a support. This ensures adequate muscle exercise for effective rehabilitation. Our team has previously designed a resistance lateral walking exercise exoskeleton [3]. Accurate gait recognition and continuous joint angle prediction are the precondition of the good control performance of the exoskeleton [4,5].

Many physiological signals have been applied to the study of gait. Among them, the surface electromyography (EMG) signal provides real-time data on muscle activity, reflecting the motion state of the human body [6]. It appears approximately 50 to 100 ms before actual movement, indicating motion intention in advance and reducing robot application delay [7]. A few studies have used EMG signal to identify human intentions and control robotic devices [8,9]. Morbidoni et al. [7] classified gait events based on sEMG signals in children with hemiplegia. Chen et al. [10] constructed a regression model linking multichannel sEMG signals with lower limb joint angles and use deep belief networks for continuous angle estimation. These studies provide a foundation for gait recognition and joint angle prediction in lateral walking using surface EMG signals.

The design of recognition algorithms is crucial for gait recognition and continuous joint angle prediction. Traditional algorithms rely on manual feature extraction and simple machine learning methods provide limited accuracy. Recently, deep learning methods substantially improve performance in these areas. We propose a graph convolutional network model (GCNM) for gait phase classification to identify the 4 leg phases between the foot and the ground. The gait data used in this study was collected by a real-time acquisition system embedded in an exoskeleton. The system included a 1,000-Hz sampling goniometer and force-sensitive resistors (FSRs) to measure hip and knee joint angles, as well as foot pressure. Data were collected from 10 healthy volunteers wearing the exoskeleton during walking experiments conducted on a force platform [11]. Wang et al. utilized wireless devices to collect surface EMG signals from 8 lower limb muscles at a 1,500-Hz sampling rate, along with footswitch signals. Additionally, an 8-camera optical system was used to capture joint angle information at a 150 Hz sampling rate. Walking experiments were performed at different speeds, and the data were synchronized. Multi-channel EMG signals were mapped to lower limb movements, and a multibranch neural network was created for joint angle estimation and gait phase recognition [12]. Wang et al. also proposed a multifeature time convolutional attention network for continuous joint angle identification and motion estimation. The data used in their study were from 2 parts: one from their own collection, which included sEMG and inertial measurement unit (IMU) angle data from 10 healthy male participants performing various upper limb movements (elbow flexion, shoulder flexion, and abduction) at 3 different speeds. The EMG signals were collected using the Noraxon Ultium EMG system with a 2,000-Hz sampling rate, and IMU angle signals had a 40-Hz sampling rate. The second part of the data was sourced from the publicly available Ninapro DB2 dataset, which includes sEMG and joint angle data from 5 participants performing wrist movements (e.g., wrist flexion and extension) at a sampling rate of 2,000 Hz [13]. Sun et al. proposed a convolutional neural network (CNN)-BiLSTM model for accurate and real-time knee joint angle prediction. The study used a publicly available benchmark dataset, which included EMG signals (1,000 Hz), IMU data (500 Hz), and knee joint kinematics data from 10 healthy participants walking on flat ground. The data were resampled to 1,000 Hz, and both EMG signals and IMU data were filtered to reduce noise for model validation [14]. These studies focus on predicting gait and joint angles for normal forward walking. Due to the obvious difference between the key gaits of forward walking and lateral walking, the algorithms designed for forward walking cannot be directly applied to lateral walking. In addition, to our knowledge, there is no research on gait recognition and joint angle prediction for lateral walking based on EMG. Therefore, the studies of normal forward walking can provide a good reference for the algorithm design of this new direction.

Various core methods of forward and other gait-type analysis are discussed, which provide a comprehensive basis for lateral gait recognition and hip joint angle prediction. Several studies have employed EMG to analyze forward gait. Cai et al. [15] recorded EMG signals from the rectus femoris, semitendinosus, gastrocnemius lateralis, and medial gastrocnemius muscles. These signals were processed using Butterworth IIR and band-stop filters. Subsequently, a linear discriminant analysis–particle swarm optimization–long short-term memory (LDA-PSO-LSTM) model was used for gait recognition. Luo et al. [16] placed EMG sensors on the tensor fasciae latae, semitendinosus, adductor longus, and vastus medialis (right leg) to collect muscle activity during walking. They utilized LSTM and multilayer perceptron (MLP) models for gait classification. Similarly, Liu et al. [17] acquired EMG data from the vastus medialis, vastus lateralis, tibialis anterior, gastrocnemius, rectus femoris, and biceps femoris muscles. They applied a 50-Hz notch filter to remove industrial frequency interference and a second-order Butterworth high-pass filter with a 10-Hz cutoff frequency. A novel metric learning-guided time-based convolutional network (ML-TCN) was proposed for gait phase identification. In a study of hemiplegic gait, Thakur and Biswas [18] employed a self-designed smartphone application. This application utilized tri-axial accelerometers and gyroscopes operating at 50 Hz, mounted at the subject’s waist. A third-order high-pass Butterworth filter with a cutoff frequency of 0.3 Hz was implemented to separate body acceleration from gravitational acceleration. A CNN-LSTM model was then used for prediction. Additionally, another study [19] employed K-nearest neighbor (KNN) to classify 4 gait patterns associated with neurodegenerative diseases. This study collected gait temporal data using insole sensors fabricated with conductive polymer layers. Two FSRs were embedded within the shoe; one at the toes and metatarsals and another under the heel. Our previous work focused on lateral gait recognition using IMU [20]. Specifically, we employed 2 lightweight, 9-axis IMUs (LPMS-B2, Alubi, China) capable of wireless data transmission. These IMUs were mounted on the shank of each subject.

**Fig. 1.**
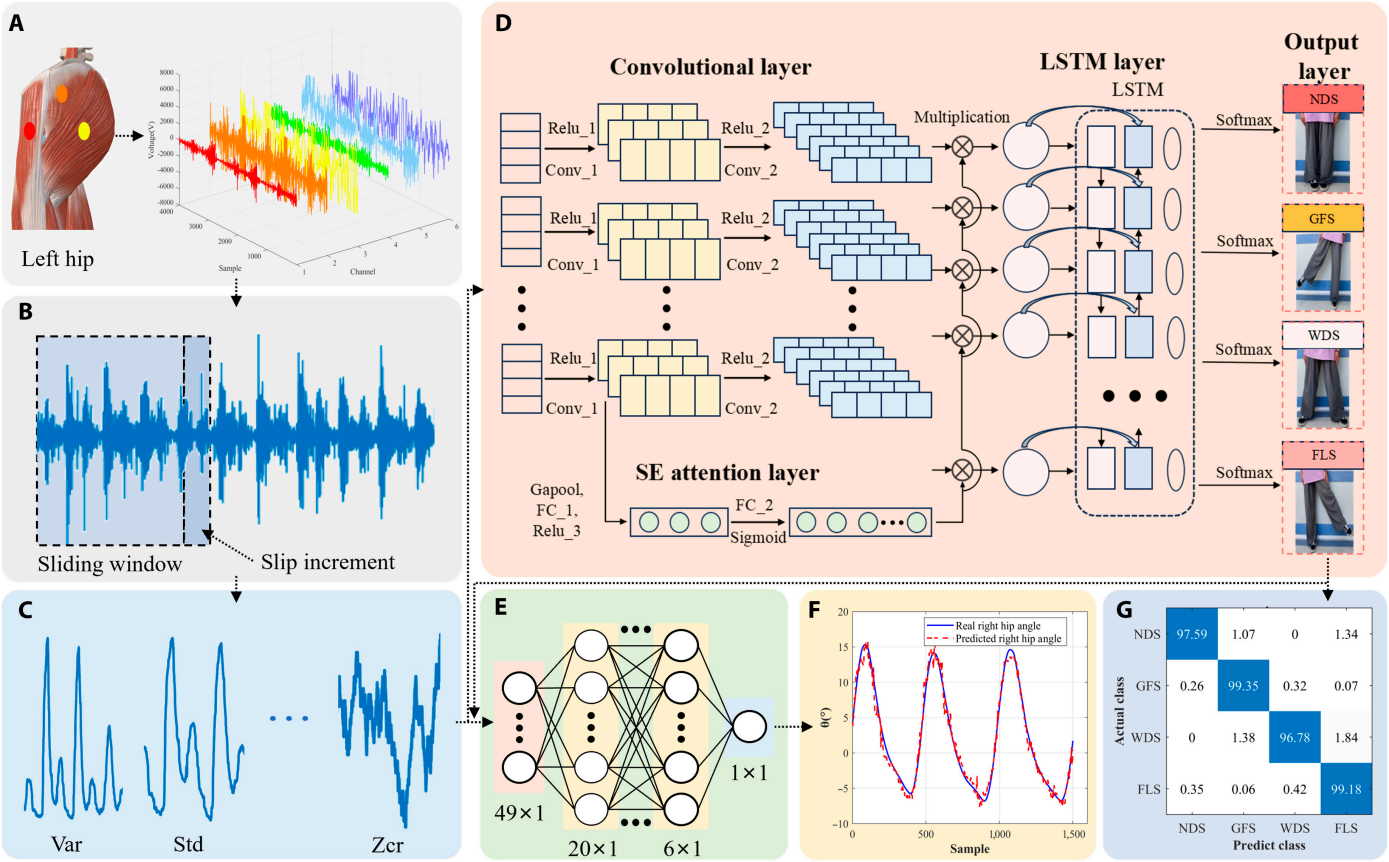
The whole process of data acquisition, preprocessing, feature extraction, model recognition, and prediction. (A) The original EMG signal is collected from the corresponding muscle. (B) The EMG signal was preprocessed and the features were extracted. (C) Extracted EMG features. (D) The “Elder Brother” module in this study consists of CNN, SEAM, and LSTM. (E) The “Younger Brother” module. (F) Prediction of continuous hip angle of “Twin Brother”. (G) Gait recognition results of lateral walking of “Twin Brother”.

Inspired by the above researches, we developed a “Twin Brother” model, as shown in Fig. 1. The model is a dual-task learning framework designed for simultaneous gait phases recognition for lateral walking and continuous hip angle prediction. It consists of 2 interconnected modules: the “Elder Brother” for gait phase recognition (classification task) and the “Younger Brother” for continuous hip angle prediction (regression task). The output of the “Elder Brother” module is used as part of the input of the “Younger Brother” module, enabling multitask collaborative learning and improving overall model performance. The main contributions of this paper can be summarized as follows:

• A “Twin Brother” combining CNN, LSTM, and the squeezing-elicited attention mechanism (SEAM) in the “Elder Brother” module for lateral walking gait phase recognition and a regression network in the “Younger Brother” module for hip joint angle estimation from EMG data. To our knowledge, this is the first study to address lateral walking gait recognition and continuous hip angle estimation with EMG signals.
• Based on the accuracy and real-time requirements of the model, the sliding window length and sliding increment were determined. Under the sliding window length of 250 ms and the sliding increment of 3 ms, the recognition accuracy of the “Twin Brother” model reaches 98.81%, which outperforms CNN-LSTM (97.24%), CNN (96.22%), LSTM (94.35%), support vector machine (SVM) (94.20%), NN (92.13%), and KNN (88.82%).
• For continuous hip joint angle estimation, compared to SVM, LSTM, and LDA, the proposed model showed better results (left leg root mean square error [RMSE]: 0.9183 ± 0.024°, *R*^2^: 0.9853 ± 0.006; right leg RMSE: 1.0511 ± 0.027°, *R*^2^: 0.9808 ± 0.008). The percentage of lateral walking gait phases was predicted, and the RMSE and *R*^2^ can reach 0.152 ± 0.014° and 0.986 ± 0.011, respectively.

## Materials and Methods

### Experimental platform

**Fig. 2.**
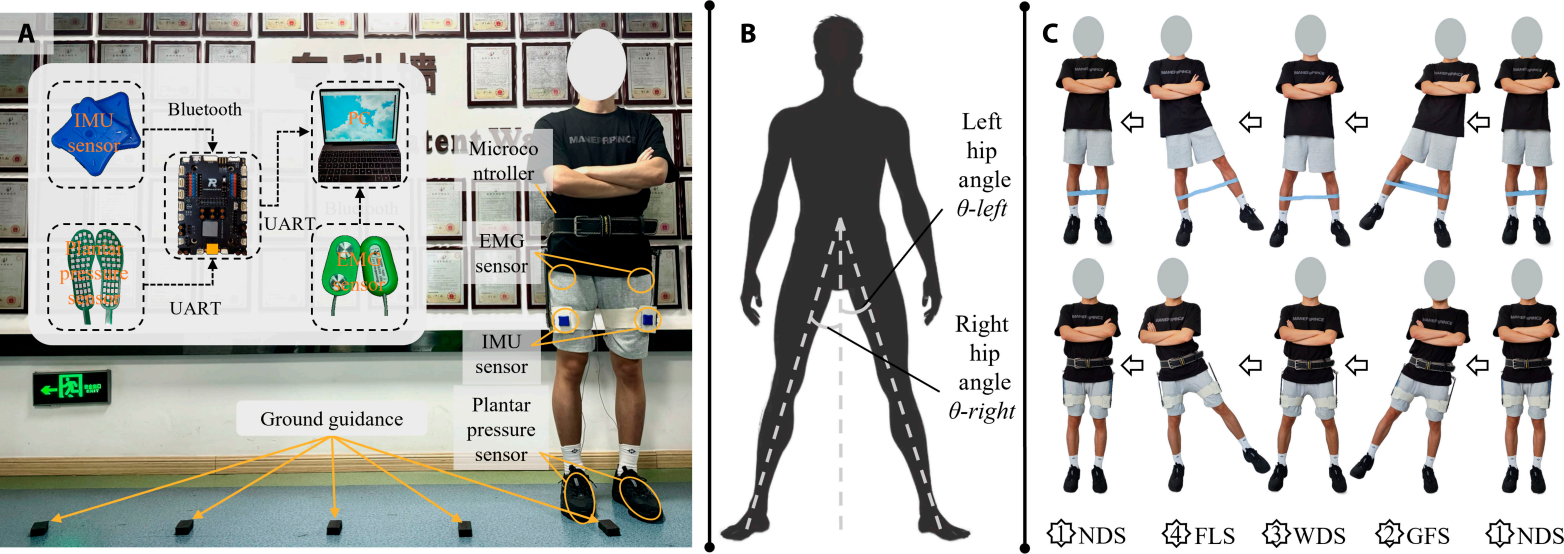
Experimental protocols, equipment, and system architecture. (A) A hip exoskeleton test platform is worn and EMG sensors, IMU, and plantar pressure sensors are placed in the lower extremities. Experimental site design and data communication between sensors. (B) The hip angle of the left and right legs measured during the experiment. (C) A complete lateral walking gait cycle using an exoskeleton instead of an elastic band.

The exoskeleton experimental platform uses 2 electric motors (GBM8008, DJI, China) for driving. Motor torque is transferred through the exoskeleton frame. The acquisition equipment includes a microcontroller, a personal computer (PC), an electromyograph, an IMU, and a plantar pressure sensor, as shown in Fig. 2A. The electromyograph (PS850, Biometrics, US) is placed on the back of the waist and uses electrodes to collect EMG signals from target muscles during lateral walking. Two IMUs (LMS-B2, Alubi, China) are placed on braces on the front of the thigh to collect human posture information during lateral walking, including hip angle (Fig. 2B). Four plantar pressure sensors are placed on the first metatarsal bone and the heel of both feet to collect pressure signals during walking, which are used as real labels for classifying gait phases. Fig. 2C illustrates a complete lateral walking gait cycle using an exoskeleton instead of an elastic band.

### Signal acquisition

Ten subjects participated in data collection and signed informed consent. The study was approved by the Medical Ethics Committee of Shenzhen Institute of Advanced Technology (SIAT-IRB-200715-H0512). The 10 subjects ranged in age from 21 to 30 years old. The weight range is from 61.3 to 92.4 kg. Height ranges from 167 to 185 cm. These selections ensured a certain degree of variability in the collected data. Before the experiment, we explained the procedure in detail. Each participant walked laterally for 30 steps at a comfortable pace (about 1.46 s/step) using ground markers placed 50 cm apart, as shown in Fig. 2A. Each subject performed 3 sets of experiments to gather enough data, with rest allowed to prevent fatigue-related data quality issues.

Data communication, shown in Fig. 2A, involves sending EMG data to a PC via Bluetooth, IMU posture information to a microcontroller via Bluetooth, and plantar pressure signals to the microcontroller via asynchronous transceiver. The microcontroller synchronizes posture and pressure data in real time and sends them to the PC. EMG and plantar pressure signals are synchronized via a wired connection for dividing EMG data into 4 gait phases.

**Fig. 3.**
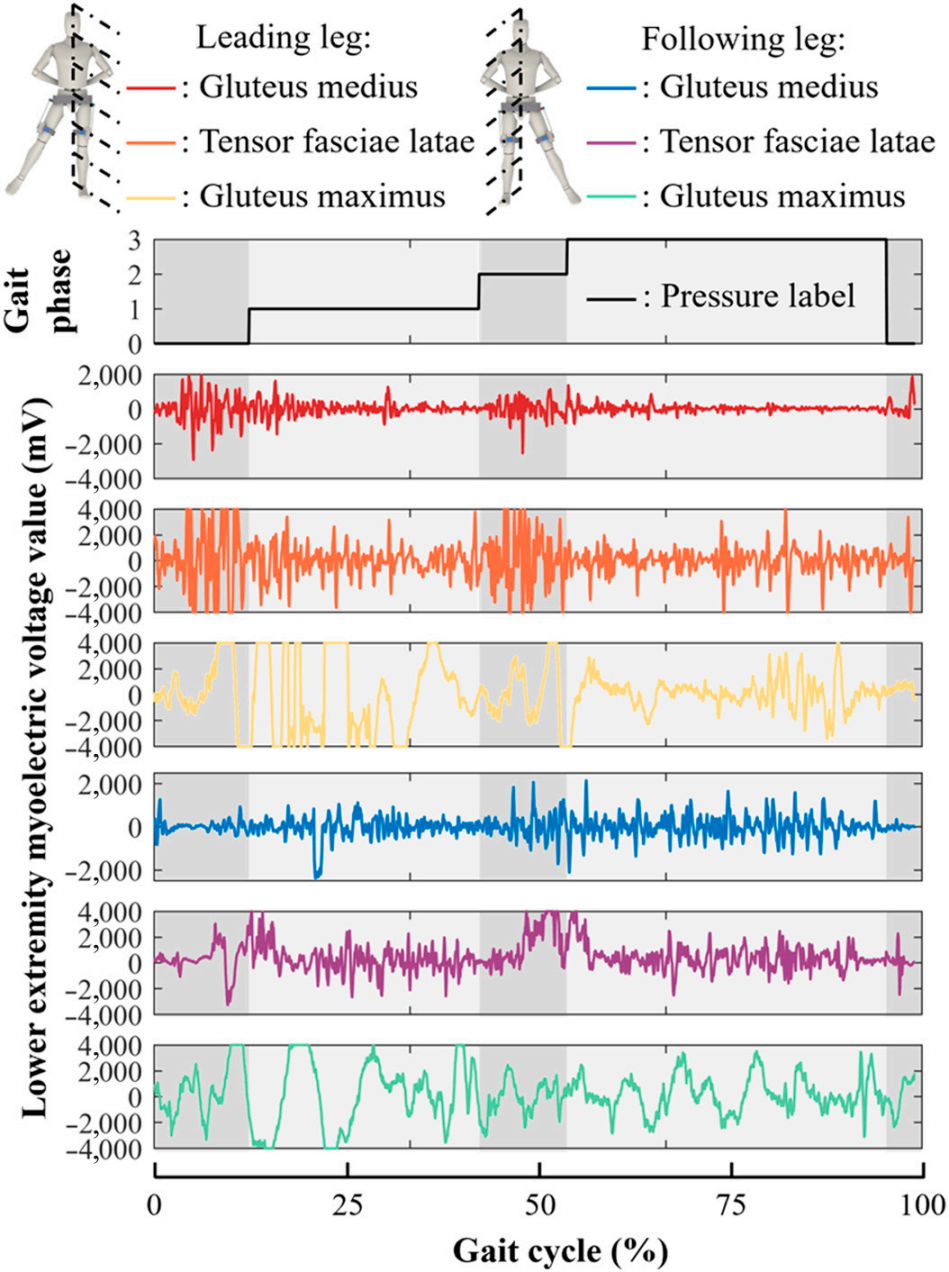
Raw signals from 6 muscles and plantar pressure signals.

EMG electrodes recorded signals from 3 muscles on each leg: gluteus medius, tensor fascia lata, and gluteus maximus, as shown in Fig. 3. Before signal collection, each muscle’s surface was cleaned with alcohol to reduce contact resistance. EMG signals were acquired at 1,000 Hz. IMUs collected hip angle signals at 200 Hz. Plantar pressure sensor data, used as the “gold standard” for dividing gait phases, were also collected at 200 Hz.

### Lateral walking gait phase division

Classifying gait phases is crucial for gait recognition. Few studies focus on lateral walking, and different teams use different divisions. In this study, we divide a complete lateral walking gait cycle into 4 phases: narrow double support (NDS), guided foot swing (GFS), wide double support (WDS), and follow leg swing (FLS). Walking to the right side is the direction of progress, with the right leg as the leading leg and the left leg as the following leg. In the first phase (NDS), the body stands relaxed with legs close together. The second phase (GFS) starts when the leading leg lifts off the ground and ends when it touches the ground again. In the third phase (WDS), the body stands relaxed with legs further apart. The fourth phase (FLS) starts when the following leg lifts off the ground and ends when it touches the ground again. Although in the NDS and WDS phases, the human body only stands statically, there is no dynamic movement. In both phases, the muscles of the legs continue to exercise weakly to maintain balance and posture. The collected signals are classified into these 2 types of gait phases. Fig. 2C also shows the complete cycle of NDS-GFS-WDS-FLS-NDS.

### Signal processing

Firstly, in order to remove the noise interference of the EMG signal, a Butterworth filter with a passband of 20 to 400 Hz is used to filter the EMG signal. Secondly, a 50-Hz notch is applied to the EMG signal to avoid power frequency interference. The collected hip angles of the left and right legs are filtered by the Kalman filter that comes with the IMU.

As mentioned earlier, plantar pressure signals are used to divide the 4 gait phases. Therefore, it is necessary to calculate the threshold triggered by the plantar pressure signal of both legs. The calculation method we used is the same as that of a previous work [11]. As shown in Table 1, after determining the threshold value, 4 gait phases were divided according to the plantar pressure state of both legs. O and F indicate the touch state and off state of the plantar pressure sensor, respectively. The triggering state of NDS and WDS gait phases is the same. We separate them according to the order in which they appear in a gait cycle.

**Table 1.** Phase division state table

Plantar pressure sensor	NDS	GFS	WDS	FLS
Left	O	O	O	F
Right	O	F	O	O

The sampling frequency of EMG does not match that of the IMU signal and plantar pressure signal. To solve the above problems, we resampled the IMU signal and plantar pressure signal. The resampled data included 6 columns of EMG signals, 1 column of gait phase labels, and 2 columns of hip angle (left and right leg).

In order to realize the continuous recognition of the EMG signal, we choose the sliding window to process the EMG signal. In addition, we explore the length and increment of the sliding window. Experiments with various window lengths and slide increments were set up. Feature extraction of EMG covered by each sliding window was carried out. Eight time domain features were extracted from the EMG signals of each channel, namely, maximum value, variance, mean absolute error, mean value, minimum value, root mean square, zero crossing times, and standard deviation. A 48-dimensional EMG feature vector is formed, including 8 features of 6 EMG channels. The feature vector finally fed into the model is constructed by combining 48 columns of EMG feature vectors, 1 column of gait phase labels, and 2 columns of hip angle (left and right leg), which is given by the following formula:

$$x=\left\{f_{1},\;f_{2},\;f_{3},\;\ldots ,\;f_{48},\;P,\;\theta_{\text{left}},\;\theta_{\text{right}}\right\}$$ (1)

Here, $f_{i},i=1,2,3,\ldots ,48$, represents the EMG features extracted from each EMG channel. P represents the gait phase. $\theta_{\text{left}}$ and $\theta_{\text{right}}$ represent the hip angle of the left and right leg measured by the IMU, respectively. After the above processing, 65,972 samples containing 51 columns of feature vectors were finally formed.

### “Twin Brother” model

Fig. 1 shows the process of data acquisition, feature extraction, model recognition, and prediction. The proposed model framework has 2 parts: the “Elder Brother” module and the “Younger Brother” module. The “Elder Brother” module recognizes the gait of lateral walking, while the “Younger Brother” module continuously estimates the hip angle. Together, these modules form our “Twin Brother” model through a specific connection.

The “Elder Brother” module in this study consists of CNN, SEAM, and LSTM. The CNN’s convolutional layer automatically learns important high-level features from the input data. LSTM, a type of recurrent neural network (RNN), is suitable for handling time series data and can remember temporal dynamics. Combining CNN and LSTM allows the model to extract high-level features and understand dynamic changes in time series. The SEAM weights the importance of each channel, optimizing the LSTM layer’s input features. This enables LSTM to model time series based on higher-quality features, improving prediction and classification accuracy.

The detailed model architecture of the “Elder Brother” module is shown in Table 2. The input layer of the “Elder Brother” module (Fig. 1D) accepts features from EMG data, typically a multidimensional time series. The input data are formatted for the convolution operation and passed to the first convolution layer for initial feature extraction. After activation by ReLU_1, the features move to the second convolution layer for more advanced feature extraction, and are then activated by ReLU_2 to learn more complex features. The CNN extracts both low-level and high-level features, identifying local patterns through its convolutional layers. The output of the first convolution layer goes to the global averaging pooling layer (gapool) to create global feature vectors. These vectors pass through 2 fully connected layers: the first outputs 1/4 of the number of channels, and the second outputs the original number of channels. The first layer’s output is activated by ReLU_3, and the second is activated by Sigmoid to generate attention weights. These weights are applied to the features from the second convolution layer through dot multiplication, allowing the model to focus on important feature channels and suppress irrelevant ones. The SEAM enhances important features and suppresses irrelevant ones. The processed features are reformatted for the LSTM layer, flattened into a one-dimensional vector. The LSTM layer models the sequence to capture time dependencies. The LSTM’s output is transformed by the fully connected layer, converted into a probability distribution by the Softmax layer, and classified. The LSTM captures dependencies between time steps to accurately identify and classify muscle activity patterns.

**Table 2.** Detailed architecture of the “Twin Brother” model

Modules	Layer	Output size	Parameters	Activation
**Elder brother**	Input Layer	48 × 1 × 1	0	-
	Conv Layer 1	46 × 1 × 32	320	ReLU
	Conv Layer 2	46 × 1 × 64	18,496	ReLU
	MaxPooling	22 × 1 × 64	0	-
	LSTM Layer	48	2,400	Tanh
	Attention Layer	48	-	Softmax
	Fully Connected 1	64	3,136	ReLU
	Fully Connected 2	16	1,040	ReLU
	Output Layer	4	68	Softmax
	Classification Layer	4	0	-
**Younger brother**	Input Layer	49 × 1	0	-
	Hidden Layer 1	20 × 1	980	ReLU
	Hidden Layer 2	12 × 1	252	ReLU
	Hidden Layer 3	6 × 1	78	ReLU
	Output Layer	1 × 1	7	-

Each hyperparameter setting of the module is shown in Table 3. The maximum number of iterations is 850, and the initial learning rate is 0.01. The learning rate decreases by a factor of 0.1 per 700 iterations, and the dataset is scrambled before each epoch. The maximum number of iterations is 850, with an initial learning rate of 0.01. The learning rate reduces every 700 iterations by a factor of 0.1, and the dataset is scrambled before each epoch. The cross-entropy loss function was used to evaluate the prediction accuracy and the Adam optimization algorithm was used for training. In this paper, the formula is as follows:

$$L=-\frac{1}{G}\sum_{i=1}^{G}\sum_{j=1}^{P}h_{\mathrm{ij}}\text{log}(H_{\mathrm{ij}})$$ (2)

Here, L is the cross-entropy loss. G is the number of samples. P is the number of categories. $h_{\mathrm{ij}}$ is the true label that sample i belongs to class j (if sample i belongs to class j, then $h_{\mathrm{ij}}=0$; otherwise, $h_{\mathrm{ij}}=1$). $H_{\mathrm{ij}}$ is the probability that the model predicts that the sample i belongs to class j. $\text{log}(H_{\mathrm{ij}})$ is the natural logarithm of the model’s prediction probability.

**Table 3.** Hyperparameters of the “Elder Brother” module

Parameters	Optimizer	Epochs	Initial learning rate	Learning rate drop factor	Learning rate drop period	Loss function
Value	Adam	850	0.01	0.1	700	Cross-entropy

As shown in Fig. 1E, the “Younger Brother” module consists of an input layer, a hidden layer, and an output layer. The input layer has 49 nodes for the EMG features and gait phase recognition results from the “Elder Brother” module. The hidden layer has 3 layers with sizes 20, 12, and 6. The output layer has one node for the hip joint angle. The detailed model architecture of the “Younger Brother” module is shown in Table 2. In the regression task, the model predicts a continuous value, using mean square error (MSE) as the loss function to measure the difference between predicted and true values. This module uses the MSE as a loss function, and its formula is as follows:

$$\text{MSE}=-\frac{1}{T}\sum_{t=1}^{T}\left(w_{t}-W_{t}\right)$$ (3)

Here, T is the number of samples. $w_{t}$ is the true value of the t sample. $W_{t}$ is the predicted value of the t sample. Algorithms 1 and 2 list the pseudocode for the “Elder Brother” and “Younger Brother” modules, respectively.


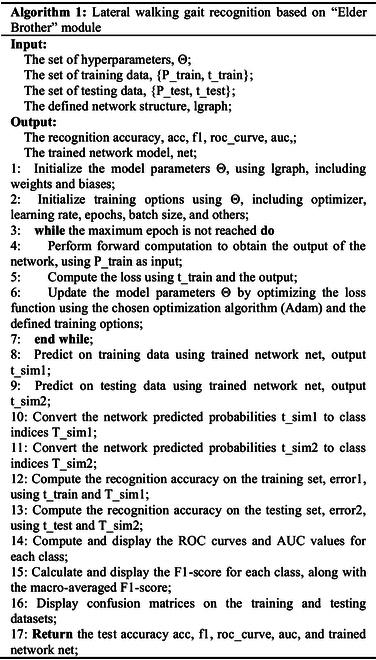



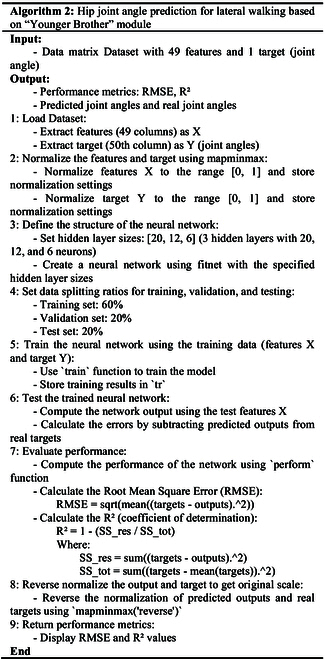


In lower limb rehabilitation robots, accurate recognition of gait phase and prediction of joint angle are essential to achieve smooth and natural assisted walking. “Elder Brother” and “Younger Brother” modules are combined to enable multitask collaboration, taking full advantage of the interconnectedness between different tasks and improving the performance and usefulness of the overall model. Different gait phases correspond to different hip joint angles during lateral walking. We combine the gait phase recognition results of the “Elder Brother” module with the original EMG into a more comprehensive feature vector, and use the feature vector as the input of the “Younger Brother” module. This helps the “Younger Brother” module predict hip angle more accurately.

### Other comparison models

As far as we know, there is no research on lateral walking gait recognition based on EMG signal. To this end, we conducted comparative experiments on some existing models commonly used in the field of gait recognition. These algorithm models include SVM, KNN, NN, CNN, LSTM, and CNN-LSTM. Cui et al. [21] used SVM fusion algorithm to improve the accuracy of gait abnormality recognition in hemiplegic patients. Angelidou and Artemiadis [22] combined KNN methods with artificial neural networks (ANNs) to accurately predict transition intentions to compliant surfaces in human gait in real time. Ling et al. [23] proposed a domain adaptive convolutional neural network (DACNN) model for gait phase recognition based on the convolutional neural network (CNN) framework. Thakur and Biswas [18] proposed a CNN-LSTM architecture for hemiplegic gait prediction. Hollinger et al. [24] compared joint level prediction performance at different gait phases based on the LSTM model. Although the above studies focus on the forward walking gait, they can provide a good reference for the recognition of lateral walking gait.

To facilitate the comparison of classical models, we define their key parameters as follows. The SVM model employed in this study utilizes the radial basis function kernel with a kernel parameter = 0.01 and a regularization parameter *C* = 1.0. For the KNN algorithm, 3 crucial factors are considered. Firstly, Euclidean distance is selected as the distance metric. Secondly, the number of neighbors K is set to 8. Finally, the majority voting method is used for decision-making. In the NN model, we design a 3-layer architecture consisting of an input layer, 2 hidden layers, and an output layer. The activation function for the hidden layers is ReLU, while the output layer employs a softmax activation function for classification. The Adam optimizer is utilized, and the cross-entropy loss function is applied for model training. For the CNN, the model includes 2 convolutional layers, each with a convolution kernel size of 2 × 1. Following the convolution layers, a max-pooling layer is applied with a pooling window size of 2 × 1. The second convolutional layer uses kernels of the same size and is activated by the ReLU function. The output layer consists of a fully connected layer with 4 neurons, and the softmax function is employed for classification. The Adam optimizer is also used, along with L2 regularization, where the regularization parameter is set to 0.0004. The initial learning rate is set at 0.001, and the learning rate follows a piecewise decay schedule, reducing by a factor of 0.1 after every 200 training epochs. In the LSTM model, an LSTM layer with 48 hidden units is utilized. This is followed by a ReLU activation layer and a fully connected layer with 4 neurons for classification. The output is produced via the softmax layer. The model is trained using the Adam optimizer with an initial learning rate of 0.01. The learning rate decays in a piecewise manner, reducing by a factor of 0.1 after every 150 epochs.

### Model evaluation index

Recognition performance: In this paper, the Confusion Matrix, ROC, area under curve (AUC), Recall, and F1-score indicators are used to show the recognition results of the model. Their relevant calculation formula is as follows:

$$CM=\left(\begin{array}{cc} \mathrm{Tp} & \mathrm{Fp}\\ \mathrm{Fn} & \mathrm{Tn} \end{array}\right)$$ (4)

$$\text{Recall}=\frac{\mathrm{Tp}}{\mathrm{Tp}+\mathrm{Fn}}$$ (5)

$$Fpr=\frac{\mathrm{Fp}}{\mathrm{Fp}+\mathrm{Tn}}$$ (6)

$$F1s=2\times \frac{\mathrm{Tp}}{2\mathrm{Tp}+\mathrm{Fp}+\mathrm{Fn}}$$ (7)

Here, Tp indicates the number of samples that were predicted to be positive but actually were. Fp indicates the number of positive cases predicted but negative cases. Tn indicates the number of samples that is predicted to be negative, and is actually negative. Fn indicates the number of samples that are predicted to be negative, but actually are positive.

Response Time: For real-time identification systems, response time is crucial. In this study, response time is the duration from receiving input data to producing output. It includes the total time for EMG data preprocessing, feature extraction, and model recognition. When choosing the sliding window increment, ensure the response time is less than the increment. This ensures the system processes and identifies the previous window’s data before new data arrive.

Root mean square error (RMSE): This index was used to evaluate the model’s performance in predicting hip joint angle. The calculation method of RMSE in this paper is as follows:

$$\text{RMSE}=\sqrt{\frac{\sum_{N=1}^{M}{\left(\theta_{M}-\theta_{P}\right)}^{2}}{M}}$$ (8)

Here, M is the total number of samples. $\theta_{M}$ is the actual hip angle measured by the IMU. $\theta_{P}$ is the hip angle predicted by the model.

R-square (*R*^2^): The value of $R^{2}$ ranges from 0 to 1, where close to 1 indicates that the model has good performance for data regression, and vice versa.

$$R^{2}=1-\frac{\sum_{N=1}^{M}{\left(\theta_{M}-\theta_{P}\right)}^{2}}{\sum_{N=1}^{M}{\left(\theta_{M}-\bar{\theta_{M}}\right)}^{2}}\times 100\%$$ (9)

Here, the meanings of M, $\theta_{M},$ and $\theta_{P}$ are consistent with those of Eq. (8). $\bar{\theta_{M}}$ is the average of the actual hip angle measured by the IMU.

## Results and Discussion

### Experiment 1: The influence of model parameter design on recognition performance

To design a high-performance “Twin Brother” model, we study the effects of sliding window length and sliding increment on recognition accuracy. Longer sliding windows capture more complete EMG cycles and features, improving recognition accuracy. However, it makes the model less responsive. Shorter sliding windows enhance temporal resolution and responsiveness but may miss critical information. Therefore, it is important to choose the right sliding window length. We compared 6 sliding window lengths from 50 to 300 ms in pre-experiments and found that 250 ms had the best effect. For further optimization, we refined the sliding window length to 220, 230, 240, 250, 260, 270, and 280 ms.

The sliding increment is also an important parameter. We set 6 sliding increments of 1, 3, 5, 10, 15, and 20 ms for each sliding window length. In real-time systems like exoskeletons, sliding increments define the interval between data windows. Smaller increments provide more overlap, capturing continuous signal changes. However, it must be greater than the system’s response time to ensure that the result can be generated within the sliding increment. Response time includes the total time for EMG data preprocessing, feature extraction, and model recognition for each sample.

**Fig. 4.**
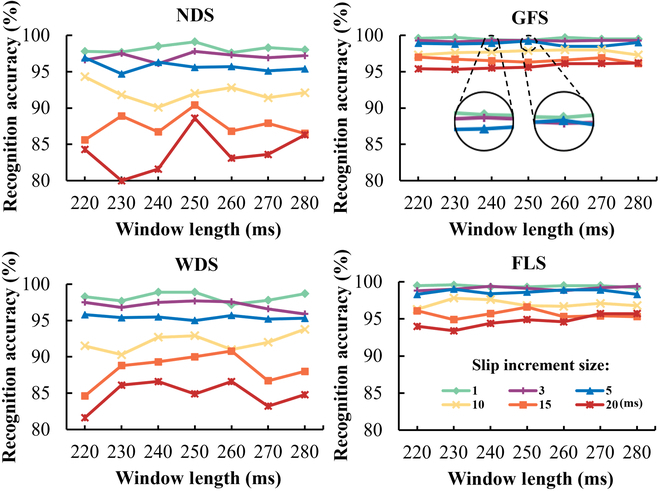
Effects of sliding window length and sliding increment on gait recognition accuracy of the “Twin Brother” model. The average recognition accuracy of 10 subjects in each gait phase was demonstrated.

The preprocessing and feature extraction time of each sample is related to the window length. The larger the window length, the longer the preprocessing and feature extraction time of each sample. The model recognition time of each sample has little correlation with the window length. Considering the range of 220 to 280 ms, 280 ms is the maximum window length. In other words, the time for each sample preprocessing and feature extraction calculated by the 280-ms window length is the longest in the range of 220 to 280 ms. A 280-ms length of data was preprocessed and EMG features were extracted. The EMG features are then put into the “Twin Brother” model for recognition. The deployment platform information of our model is as follows: hardware [NVIDIA GeForce RTX 4060, 12th Gen Intel (R) Core (TM) i5-12490F and 32 GB RAM] and software (MATLAB R2022a). As shown in Table 4, we provide the calculation speed of the model on the corresponding deployment platform. Specifically, we calculated 4 metrics: preprocessing and feature extraction, model recognition, response time, and samples per second. Preprocessing and feature extraction refers to the time of preprocessing and feature extraction from each input sample. Model recognition refers to the time required for a model to recognize each input sample. As mentioned above, response time is the sum of preprocessing and feature extraction and model recognition. Samples per second indicates the number of samples recognized by the model per second. After calculation, the average time of EMG data preprocessing and feature extraction for each sample was 2.012 ms, the average time of model identification for each sample was 0.873 ms, the response time of the whole experiment was 2.885 ms, and the number of samples recognized by the model per second was 1,145. Therefore, the slip increment needs to be greater than 2.885 ms. As can be seen from Fig. 4, when the slip increment increases from 1 to 20 ms, the recognition accuracy of the 4 gait phases decreases to varying degrees. The sliding increment of 3 ms is a suitable choice, which can maintain a high recognition accuracy and meet the real-time demand greater than the response time of 2.885 ms. In the 3-ms sliding increment, NDS performs better in the sliding window length of 230, 250, and 260 ms. The sliding window length between 240 and 280 ms in GFS is similar. The sliding window length of WDS is 240, 250, and 260 ms. The sliding window length of FLS is 240, 250, 270, and 280 ms. It can be seen that when the slip increment is 3 ms and the slide window length is 250 ms, the recognition effect of the 4 gait phases is both better. Therefore, we chose to conduct other experiments using a parameter configuration with a sliding window length of 250 ms and a sliding increment of 3 ms.

**Table 4.** The computation speed for each part

Type	Preprocessing and feature extraction (ms)	Model recognition (ms)	Response time (ms)	Samples per second
Computation speed	2.012	0.873	2.885	1,145

### Experiment 2: Experiment and comparison of lateral walking gait recognition based on the “Twin Brother” model

Based on the EMG data of the same 10 subjects, this experiment explored the gait recognition accuracy of the models. We compare the “Twin Brother” model in this paper with 5 classical models. The EMG data of 10 subjects were divided into a training set and a test set in a ratio of 7:3. The method of 10× cross verification is adopted.

**Fig. 5.**
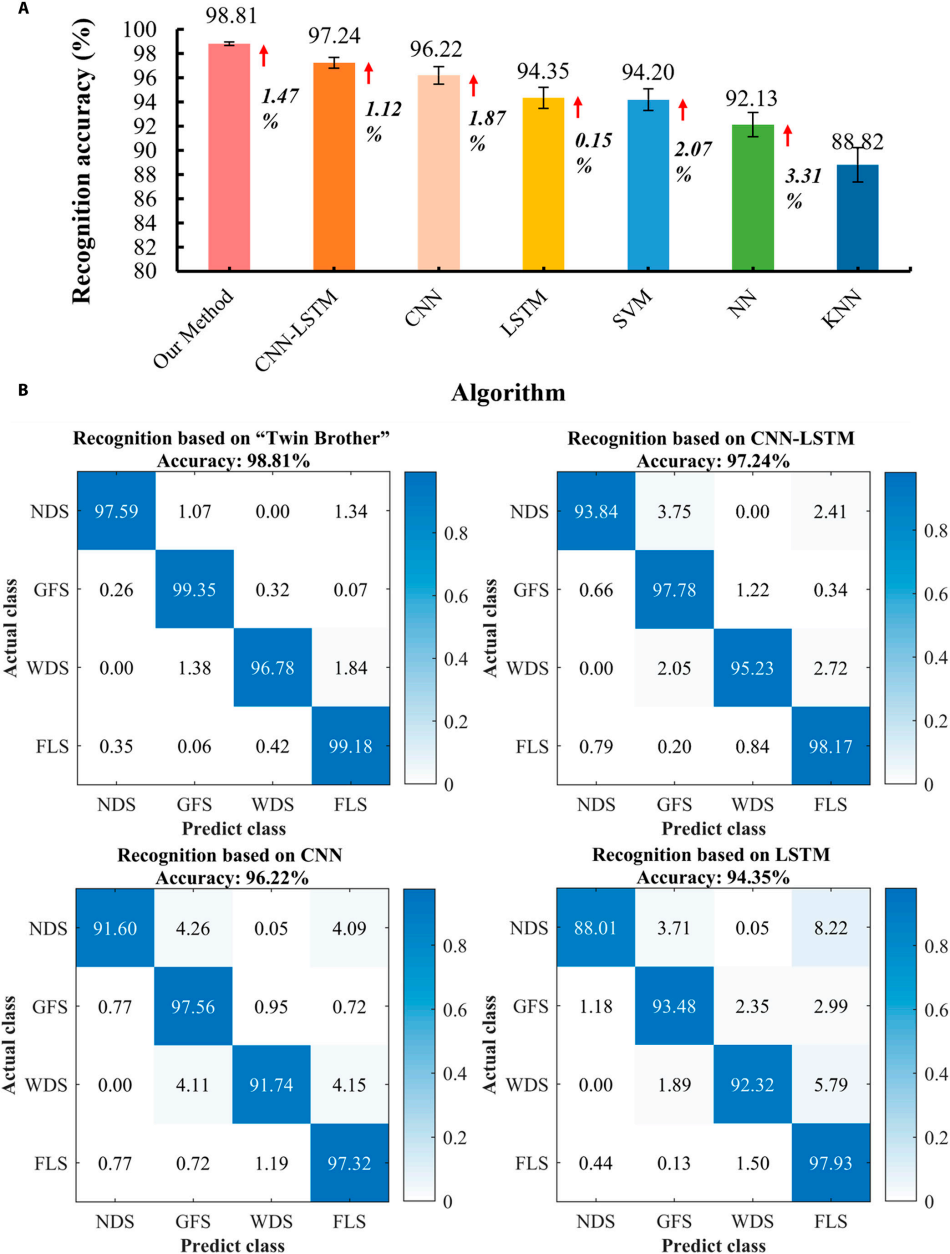
Gait recognition results of different models for lateral walking. (A) Based on the same EMG data, the gait recognition accuracy of the proposed method is compared with other classical models (CNN-LSTM, CNN, LSTM, SVM, NN, and KNN). (B) The recognition results of 4 gait phases (NDS, GFS, WDS, and FLS) by different deep learning models are presented in detail in the form of confusion matrix.

Fig. 5A shows the total recognition accuracy of different models for 4 gait phases in the form of a bar chart. The mean and standard deviation (mean ± SD) of recognition accuracy of different models were 98.81% ± 0.14% (our method), 97.24% ± 0.46% (CNN-LSTM), 96.22% ± 0.72% (CNN), 94.35% ± 0.88% (LSTM), 94.20% ± 0.91% (SVM), 92.13% ± 1.01% (NN), and 88.82% ± 1.42% (KNN). It can be seen that our method is higher than that of other models. Specifically, as shown in Fig. 5B, we use confusion matrix to show the gait recognition results of 3 deep learning models. Table 5 shows the confusion matrix of gait phase recognition accuracy based on SVM, NN, and KNN. From Fig. 5B and Table 5, the average recognition accuracy of the other 6 recognition models (CNN-LSTM, CNN, LSTM, SVM, NN, and KNN) for NDS in the gait phase was 93.84%, 91.6%, 88.01%, 89.39%, 79.74%, and 70.33%, respectively. The average recognition accuracy of GFS in the gait phase was 97.78%, 97.56%, 93.48%, 96.58%, 95.91%, and 93.55%, respectively. The average recognition accuracy of WDS in the gait phase was 95.23%, 91.74%, 92.32%, 86.43%, 84.34%, and 76.62%, respectively. The average recognition accuracy of FLS in the gait phase was 98.17%, 97.32%, 97.93%, 95.35%, 93.27%, and 92.01%, respectively. The accuracy of the “Twin Brother” model for the 4 gait phases of NDS, GFS, WDS, and FLS was 97.59%, 99.35%, 96.78%, and 99.18%, respectively, which exceeded 95% and exceeded other models. The results show that the method proposed in this paper has a good recognition effect in the total recognition accuracy of gait phase and the recognition accuracy of each single gait phase. To enhance the persuasiveness of our findings, we have included the results of paired *t* tests. Specifically, we conducted paired *t* tests on the recognition accuracy results of all models to assess the statistical significance of performance differences between the “Twin Brother” model and other models. Through these calculations, the *P* values for DAE-LSTM, CNN-LSTM, CNN, LSTM, SVM, NN, and KNN were found to be 0.021, 0.033, 0.026, 0.041, 0.028, 0.037, and 0.018, respectively. These results indicate that the “Twin Brother” model demonstrates statistically significant improvements in recognition accuracy compared to all other models (*P* < 0.05).

**Table 5.** Confusion matrix of gait phase recognition accuracy based on SVM, NN, and KNN (%)

Models		SVM (Total: 94.20)					NN (Total: 92.13)					KNN (Total: 88.22)			
		NDS	GFS	WDS	FLS		NDS	GFS	WDS	FLS		NDS	GFS	WDS	FLS
		Predict class					Predict class					Predict class			
NDS	Predict class	89.39	5.52	0	5.09		79.74	11.31	0.11	8.84		70.33	16.1	0.46	13.11
GFS		1.32	96.58	1.74	0.36		1.78	95.91	1.49	0.82		2.08	93.55	2.35	2.01
WDS		0	5.53	86.43	8.04		0.04	7.41	84.34	8.21		0	10.73	76.62	12.65
FLS		1.31	0.82	2.51	95.35		2.28	1.17	3.28	93.27		1.67	2.94	3.39	92.01

**Fig. 6.**
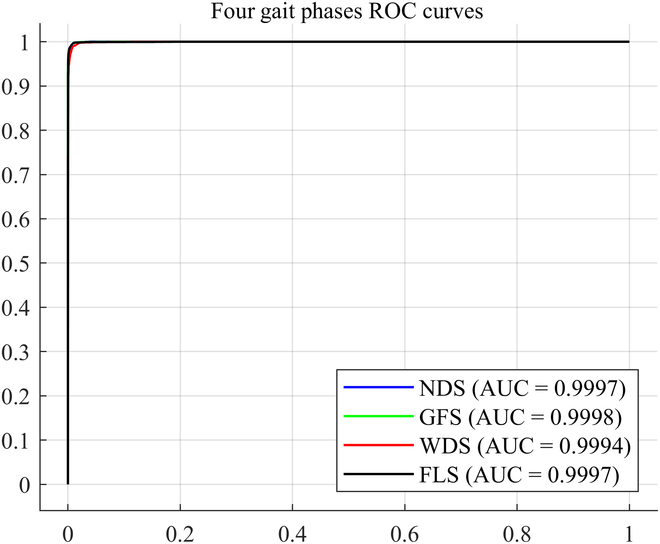
The ROC curve of the "Twin Brother" model for 4 gait phases.

To provide a more comprehensive and accurate evaluation of the recognition performance of the “Twin Brother” model, we plotted the model’s ROC curves on the test set and computed the AUC, Recall, and F1-score for each gait phase. As shown in Fig. 6, the ROC curves for the 4 gait phases on the test set demonstrate excellent performance, with high AUC values for each phase. These values indicate that the model effectively differentiates between the positive and negative classes for each gait phase. In Table 6, we present the average values for AUC, Recall, and F1-score across the 4 gait phases. Specifically, for the NDS phase, the model achieved average values of 0.9997 (AUC), 0.9775 (Recall), and 0.9713 (F1-score). Similarly, for the GFS, WDS, and FLS phases, the average values of these metrics were as follows: 0.9998, 0.9994, and 0.9996 for AUC; 0.9877, 0.9732, and 0.9919 for Recall; and 0.9914, 0.9693, and 0.9902 for F1-score. The overall average values across all 4 gait phases for each metric were 0.9996 for AUC, 0.9826 for Recall, and 0.9805 for F1-score. These results underscore the robustness and high performance of the “Twin Brother” model in recognizing the 4 lateral walking gait phases.

**Table 6.** Mean recognition performance based on the “Twin Brother” model

Index	NDS	GFS	WDS	FLS	Mean
AUC	0.9997	0.9998	0.9994	0.9997	0.9996
Recall	0.9775	0.9877	0.9732	0.9919	0.9826
F1-score	0.9713	0.9914	0.9693	0.9902	0.9805

To verify the reliability of the results, we compared them with other recent studies in Table 7. Due to the lack of lateral walking gait recognition studies based on EMG, we compared a few forward walking gait recognition studies based on EMG. Nevertheless, they can still provide a reference to verify the reliability of the results. Based on EMG signals, the LAD-PSO-LSTM model was proposed in [15] to recognize 7 gait phases, and the recognition accuracy was 94.90%. Studies [11,16,17] are similar to our study in that they all recognize 4 gait phases based on EMG signals. In Ref. [7], the author trained an MLP model to recognize the 2 gait phases, and the average recognition accuracy was 97% for intra-subject. In the study by Mengarelli et al. [19], the author used the KNN model to identify 4 categories based on the pressure insole signal, and the overall recognition accuracy was 94.84%. Di Nardo et al. [25] used an MLP architecture to estimate gait events during walking, with an average classification accuracy of 96.10% in terms of heel-strike and toe-off. All of them achieved a good gait recognition effect. Similar to their results for forward walking, we have obtained similar recognition accuracy for lateral walking gait recognition. In addition, compared with our previous recognition results based on IMU [20,26], the EMG research in this paper further improves the recognition accuracy. In addition to recognition accuracy, several studies have reported on the speed of models for gait recognition. Xu and Wang [27] used a quadratic discriminant analysis (QDA) model to recognize forward walking gait, achieving a recognition time of 5.36 ms for a single feature vector. In our previous work, we used Urban Buildings Indexing (UBI) to recognize lateral walking gait, with an average recognition time of 5.7 ms for each feature vector [20]. In this paper, our “Twin Brother” model has a response time of 2.885 ms. Similar to these studies, the response speed of the model in this paper is also fast, demonstrating good potential for practical application.

**Table 7.** Comparison of gait phase recognition performance between the “Twin Brother” model and other studies

Reference	Method	Signal type	Gait phase number	Accuracy
[16]	LSTM and MLP	EMG	4	94.10%
[26]	SDA-NN-ML	IMU	4	94.56%
[19]	KNN	Pressure insole	4	94.84%
[15]	LDA-PSO-LSTM	EMG	7	94.90%
[25]	MLP	EMG	2	96.10%
[17]	ML-TCN	EMG	4	96.22%
[20]	UBI	IMU	4	96.64%
[7]	MLP	EMG	2	97.00%
[11]	GCNM	EMG	4	97.43%
This work	“Twin Brother”	EMG	4	98.81%

### Experiment 3: Continuous prediction and comparison of hip joint angle of both legs based on “Twin Brother” model

Based on the collected EMG data and the hip angle, the “Twin Brother” model proposed in this paper also has the function of predicting the hip angle. As far as we know, there are no relevant studies on the prediction of hip angle in lateral walking based on EMG. Many studies using EMG to continuously estimate joint angle are focused on forward walking. Wang et al. [28] introduced a TCN algorithm based on the sensing system of acoustic and bioelectrical signals to predict the angle of the contralateral lower limb joint during human motion. Zhou et al. [29] proposed a multistream signal fusion strategy based on knowledge tracking (MSKT) to reconstruct multistream signals such as surface EMG into new features to continuously estimate the angle of lower limb joints. Zhong et al. [30] propose a muscle-synergistically driven adaptive network fuzzy reasoning system approach to predict continuous knee movements. Inspired by this, we predict the hip angle of lateral walking based on myoelectric characteristics. To provide more feature information, we combine the output of the “Elder Brother” module (gait phase recognition result) and the input (EMG feature) into a new feature vector. This new feature vector is used as the input of the “Younger Brother” module, and the hip angle is the output of the “Younger Brother” module.

**Fig. 7.**
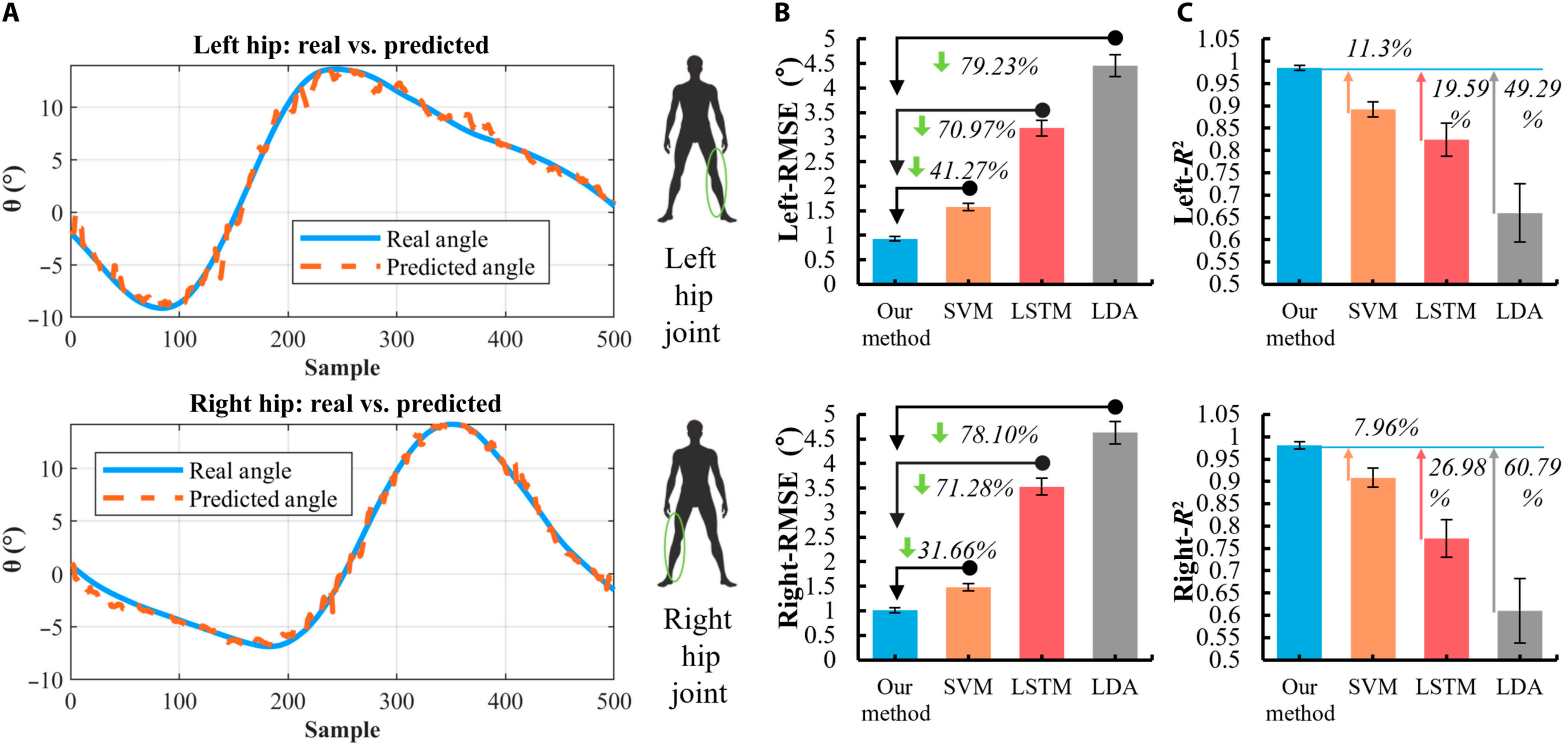
Prediction and comparison of hip joint angle in lateral walking. (A) The left and right hip angles measured by the IMU and predicted by the “Twin Brother” model. (B) The RMSE predicted by the “Twin Brother” model was compared with other predictions based on classical EMG features. (C) The *R*^2^ predicted by the “Twin Brother” model and other models.

Fig. 7A shows the angle predicted by the “Twin Brother” model versus the angle measured by the IMU. It can be seen that the proposed model has good tracking performance and prediction accuracy for hip joint angle during lateral walking. Fig. 7B and C show the prediction performance of the “Twin Brother” model compared to other predictions based on some classical EMG features. Based on EMG data from the same 10 subjects, the hip angle RMSE (mean ± SD) predicted by the “Twin Brother” model was 0.9183° ± 0.024° (left leg) and 1.0511° ± 0.027° (right leg). Similarly, the hip joint angle prediction performance of SVM, LSTM, and LDA models was 1.5755° ± 0.057°, 3.1864° ± 0.198°, and 4.4533° ± 0.297° (left leg) and 1.4842° ± 0.053°, 3.5315° ± 0.204°, and 4.6314° ± 0.351° (right leg), respectively. The hip angle *R*^2^ (mean ± SD) predicted by the “Twin Brother” was 0.9853 ± 0.006 (left leg) and 0.9808 ± 0.008 (right leg). The hip angle *R*^2^ predicted by SVM, LSTM, and LDA models was 0.8923 ± 0.017, 0.8238 ± 0.037, and 0.6614 ± 0.065 (left leg) and 0.9085 ± 0.021, 0.7723 ± 0.042, and 0.6121 ± 0.072 (right leg), respectively. As you can see, the “Twin Brother” model in this study achieves lower RMSE and higher *R*^2^ than the other models. To further validate these performance differences, we conducted paired *t* tests on the hip angle prediction results. These tests indicated that the “Twin Brother” model demonstrates statistically significant improvements in prediction accuracy compared to the SVM, LSTM, and LDA models, with *P* values less than 0.05. Specifically, these *P* values were 0.018, 0.041, 0.036, and 0.028, respectively. The results show that the “Twin Brother” model in this paper has good predictive performance and application potential in predicting hip joint angle during lateral walking.

Table 8 summarizes the performance of our method compared with the most advanced forward walking studies, showing that our method achieves advanced performance in lateral walking. The DBN model only uses the features extracted from the surface EMG signal to predict the angle of lower limbs [10]. In addition, studies [28,29] have combined EMG and other signals to predict hip angle, which is better than only using EMG signals. Therefore, the use of EMG signals to estimate joint angle deserves further attention, and our method provides strong support and new ideas for this research direction.

**Table 8.** Comparison of hip joint angle prediction performance between the “Twin Brother” model and other models

Reference	Method	Signal type	Performance (hip joint)
[10]	DBN	EMG	RMSE = 3.58°
[28]	TCN	EMG and VAG	RMSE = 1.82°
[29]	MSKT	FMG, EMG, and VAG	RMSE = 1.49°
This work	“Twin Brother”	EMG	RMSE (left) = 0.9183°, RMSE (right) = 1.0511°, *R*^2^ (left) = 0.9853, *R*^2^ (right) = 0.9808

In the above experiments, we observed the potential of the “Twin Brother” model in predicting hip joint angles. To further investigate, we conducted online experiments to explore the real-time prediction capability of the “Twin Brother” model for hip joint angles during lateral walking. We used the model trained in the offline experiments for the experiment. The subject was instructed to wear the same sensors for real-time data collection while performing a lateral walking task. During the experiment, EMG, IMU, and foot pressure signals were collected to provide the necessary input data for the model. The “Twin Brother” model then predicted the left and right hip joint angles in real time based on the input data.

**Fig. 8.**
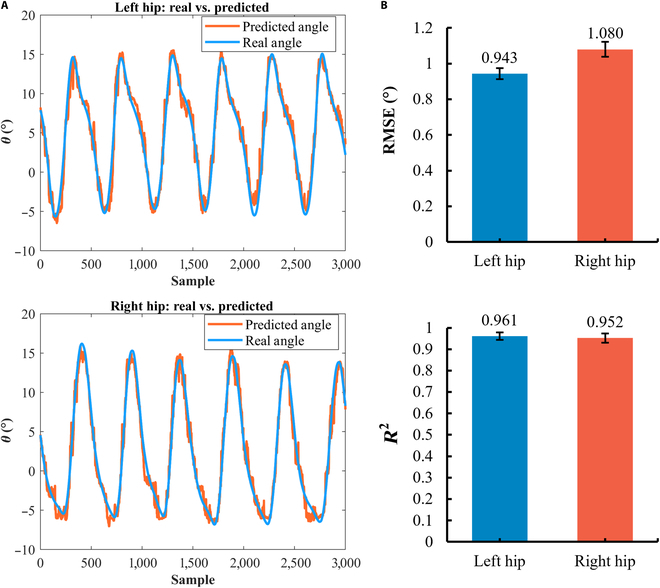
Prediction of hip joint angle in online experiment. (A) The left and right hip angles measured by the IMU and predicted by the “Twin Brother” model in online experiment. (B) The RMSE and *R*^2^ predicted by the “Twin Brother” model.

To quantify the model’s prediction performance, we also used 2 evaluation metrics: RMSE and *R*^2^. Fig. 8A shows the real hip angles and the predicted angles for both the left and right legs. It can be seen that the model effectively tracks the real angle curves. Specifically, Fig. 8B quantifies the tracking performance. The RMSE (mean ± SD) for the left leg was 0.943° ± 0.031°, and for the right leg, it was 1.080° ± 0.042°. The *R*^2^ (mean ± SD) values were 0.961 ± 0.018 for the left leg and 0.952 ± 0.021 for the right leg. These results further highlight the ability of the “Twin Brother” model based on EMG data to predict hip angle and demonstrate its practical application potential.

### Experiment 4: Percentage prediction of lateral walking gait phases based on EMG signals

Similar to forward walking, percentage prediction of gait phases during lateral walking is also important for exoskeleton control [31,32]. Lee et al. [33] used the data measured by the IMU to continuously estimate gait phases during walking. Based on the EMG signals of lateral walking, we also discuss the prediction of the percentage of gait phase. Each gait phase divided by the plantar pressure signal is mapped to a range of [0% to 100%]. Then, the mapped data are used as the output for “Twin Brother” model training and prediction. The input features of the model are the same as those of the hip angle prediction task, namely, the outputs (gait recognition results) and inputs (EMG features) of the “Elder Brother” module.

**Fig. 9.**
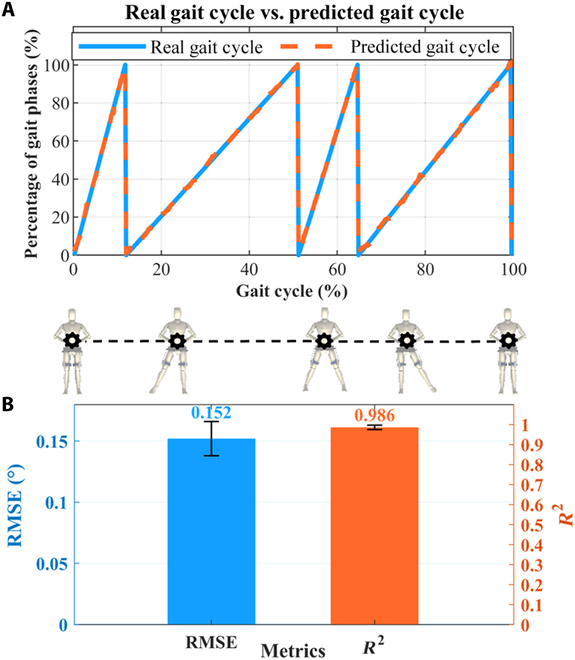
Percentage prediction of lateral walking gait phases based on EMG signals. (A) The real gait cycle percentage curve and the predicted gait cycle percentage curve based on the “Twin Brother” model. (B) RMSE and *R*^2^ of the model for gait phases percentage prediction.

Fig. 9 shows the percentage prediction results of lateral walking gait phases based on EMG signals. From Fig. 9A, we can see that the predicted gait cycle percentage curve has a high coincidence degree with the real curve. Fig. 9B shows the quantified prediction results. The predicted RMSE and *R*² (mean ± SD) were 0.152° ± 0.014° and 0.986 ± 0.011, respectively. It will provide a reference for the future research of gait phase percentage prediction in lateral walking.

### Experiment 5: Research on advanced gait phase recognition ability of “Twin Brother”

In this experiment, based on the “Twin Brother” model trained in Experiment 2, we investigated its ability to perform advanced gait phase recognition. Specifically, during the model testing, we adjusted the input feature vectors and their corresponding labels. For the experiment without advanced gait phase recognition, the input feature vectors correspond to the labels of the current sliding window. In contrast, for the advanced recognition task, we modified the input feature vectors so that their corresponding labels represented the gait phases of future sliding windows. This setup enabled the model to predict the gait phases of future sliding windows based on the current sliding window’s data. We varied the number of sliding increments to explore how the model recognizes gait phases over different time intervals. For example, when the number of sliding increments is 1, the feature vector of the model test input is current, and the input label is the corresponding one after the sliding window slides one increment.

We conducted tests with 6 different sliding increment values to assess the model’s prediction accuracy for varying time horizons. Table 9 presents the model’s performance with different sliding increments. As the sliding increment increases, the model’s performance gradually diminishes, which is consistent with the intuition that predicting future events becomes more challenging as the time window extends. Specifically, when the model recognized advance 2 increment, it achieved an accuracy of 98.13%, an AUC of 0.9993, a Recall of 0.9735, and an F1-score of 0.9727. This indicates that the model can robustly and accurately identify gait phases with good performance in short-term prediction tasks.

**Table 9.** Mean recognition performance based on different advance sliding increments

Sliding increments	0	2	5	10	15	20
Accuracy	98.81%	98.13%	95.92%	91.96%	88.59%	84.52%
AUC	0.9996	0.9993	0.9960	0.9856	0.9712	0.9471
Recall	0.9826	0.9735	0.9429	0.8868	0.8365	0.7800
F1-score	0.9805	0.9727	0.9416	0.8850	0.8357	0.7792

**Fig. 10.**
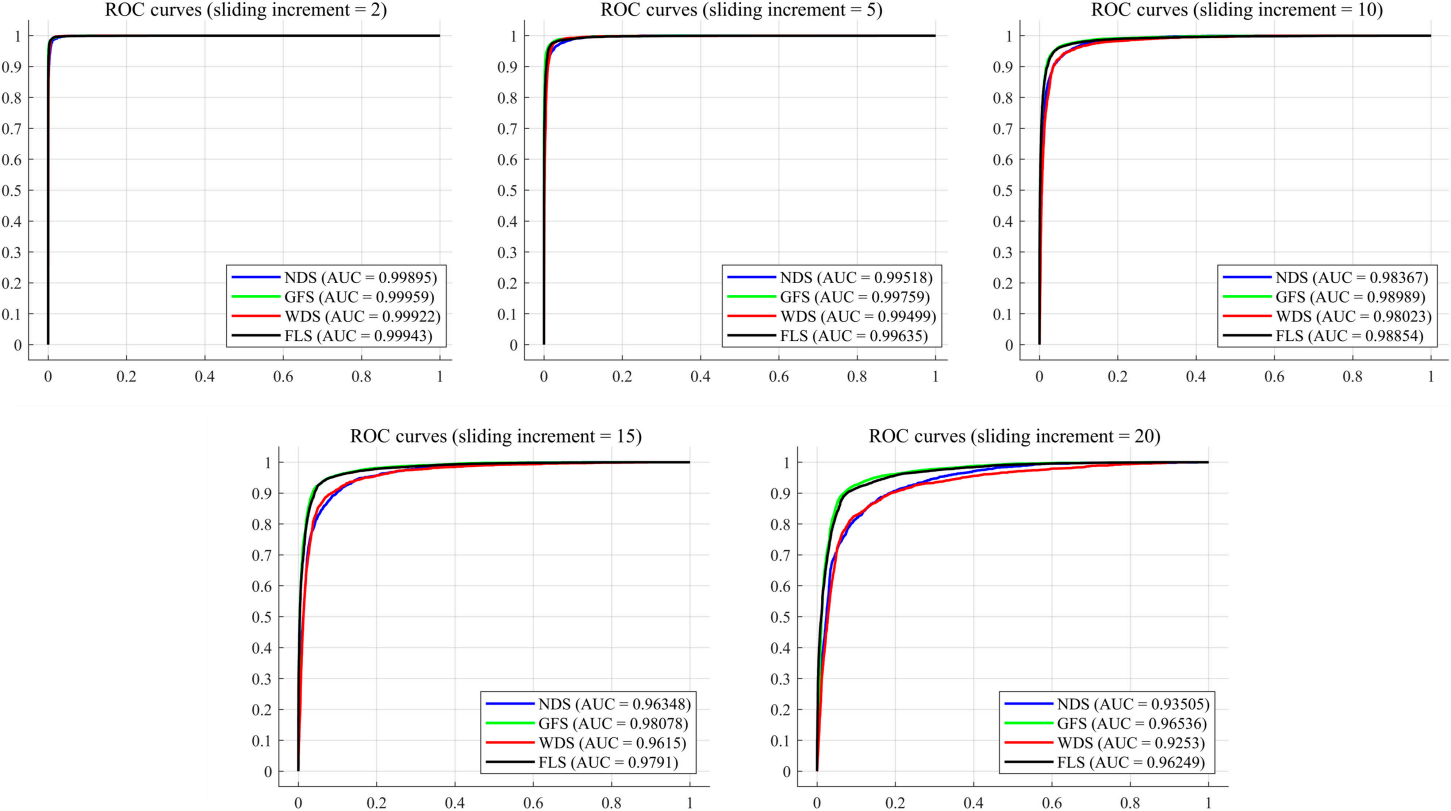
The ROC curve of “Twin Brother” under different advance sliding increments.

However, when recognizing advance 5 sliding increments, the accuracy dropped to 95.92%, accompanied by an AUC of 0.9960, a Recall of 0.9429, and an F1-score of 0.9416. Although the accuracy still remained high, there was a noticeable reduction in the model’s performance. As the sliding increment was extended further to 10, 15, and 20, the recognition accuracy continued to decline, reaching 91.96%, 88.59%, and 84.52%, respectively. Corresponding AUC values for these increments were 0.9856, 0.9712, and 0.9471, while Recall values were 0.8868, 0.8365, and 0.7800, respectively. The F1-scores also exhibited a similar downward trend, with values of 0.8850, 0.8357, and 0.7792. Notably, when predicting 15 sliding increments, the accuracy dropped below 90%, signaling an obvious reduction in model performance. Fig. 10 visually illustrates this trend through ROC curves, showing the model’s performance across the 4 gait phases under different advance sliding increments.

These findings highlight a trade-off between prediction horizon and model performance, with the model performing optimally for short-term (2 to 5 sliding increment) recognitions and showing a gradual decline in effectiveness as the prediction horizon extends. In particular, the decreasing AUC, Recall, and F1-score values reinforce the model’s diminishing ability to maintain accurate gait phase recognitions over longer time periods.

### Experiment 6: Evaluation of hip joint angle prediction performance under different proportion of test sets

To more comprehensively evaluate the model’s continuous prediction performance across varying signal lengths, we conducted a further investigation building upon Experiment 3. Specifically, we leveraged the model trained in Experiment 3 to assess its performance on test datasets of different lengths. To simulate these varying signal lengths, we selected the continuous 10%, 20%, 30%, 40%, and 50% of the recordings from all subjects in the original dataset as independent test sets. This approach allowed us to construct test signals of different lengths, enabling us to evaluate the model’s predictive performance under different signal length conditions and investigate whether the model experiences a performance degradation when predicting longer signals. The model’s prediction performance was quantified using the RMSE and *R*^2^ as evaluation metrics. This experiment aims to validate the stability and reliability of the model’s predictive capabilities when faced with input data of varying lengths in real-world applications.

As shown in Table 10, when the signal length proportion was 10%, the average RMSE for the left hip joint angle prediction was 1.0106, and the average *R*^2^ was 0.9835. As the proportion increased to 20%, 30%, 40%, and 50%, the average RMSE values for the left hip joint angle prediction were 1.0003, 1.0074, 1.0038, and 1.0054, respectively, and the average *R*^2^ values were 0.9827, 0.9813, 0.9823, and 0.9819, respectively. For the right hip joint angle prediction, at a 10% signal length proportion, the average RMSE was 1.0426, and the average *R*^2^ was 0.9803. When the proportion increased to 20%, 30%, 40%, and 50%, the average RMSE values for the right hip joint angle prediction were 1.0390, 1.0623, 1.0404, and 1.0445, respectively, and the average *R*^2^ values were 0.9797, 0.9794, 0.9804, and 0.9801, respectively. These results indicate that the model’s performance demonstrates relative stability across the different signal length proportion. Specifically, the RMSE values for both left and right hip joint angle predictions remained consistently low across the various signal length proportions investigated. While minor fluctuations in RMSE were observed, the magnitude of these variations was small. This indicates that the model’s predictive performance is not substantially affected by alterations in signal length. Furthermore, the observed *R*^2^ for both left and right hip joint angles demonstrated consistently high values, exhibiting no obvious changes across the different signal length proportions. This further corroborates the notion that the model effectively captures the underlying patterns and relationships within the data, irrespective of signal length. Notably, in practical applications, the required signal length for hip joint angle prediction may vary depending on the specific use case. This experimental finding validates the applicability of the model in different practical application scenarios.

**Table 10.** Mean prediction performance under different proportional signal lengths

Proportion/%	10	20	30	40	50
Left-RMSE/(°)	1.0106	1.0003	1.0074	1.0038	1.0054
Left-*R*^2^	0.9835	0.9827	0.9813	0.9823	0.9819
Right-RMSE/(°)	1.0426	1.0390	1.0623	1.0404	1.0445
Right-*R*^2^	0.9803	0.9797	0.9794	0.9804	0.9801

In addition to normal forward walking and lateral walking, some people may have a combination of forward walking and lateral walking due to disease. Although the “Twin Brother” model based on EMG, proposed in this paper, primarily verifies gait recognition and hip joint angle prediction for lateral walking, its principle is not limited to “standard lateral walking”. It can also be extended to a mixed walking mode that combines forward and lateral walking, allowing for the identification and monitoring of lateral components. The EMG signals generated by different walking modes (such as pure forward walking, pure lateral walking, and a mixture of both) reflect the contraction and relaxation characteristics of the muscles in the gait of a particular walking mode. When we carry on forward and lateral joint walking at a certain speed and a certain stride length, the collected EMG signals often still show a certain regularity. In other words, even in mixed walking modes, there are relatively stable myoelectric features that can be captured by the model. These stable myoelectric signatures also contain information about the “lateral walking component”. In the process of data annotation or training, as long as this part of the signal is matched with the label corresponding to “lateral walk”, the model can identify and monitor the horizontal component. The “Elder Brother” module (based on CNN, SEAM, and LSTM) can conduct multidimensional modeling of temporal EMG data, which can not only extract spatial features but also capture the law of gait change over time. For the forward lateral mixed gait, the “Elder Brother” module can identify the corresponding lateral component by matching the regular signal with the corresponding gait label in the lateral component and by the dynamic change of the EMG signal. Similarly, the regular signal is matched with the hip angle label corresponding to the lateral component and lateral walking component identified by the “Elder Brother” module and used for the training of the “Young Brother” module. In the prediction stage, the “Younger Brother” module can predict the hip angle by using the EMG characteristics and the lateral component gait phase identified by the “Elder Brother” module. In this way, even if the forward part of the entire walk is included, the model can also “identify” and “monitor” the lateral part during training, achieving more flexible application effects. In subsequent work, if more forward and lateral combined walking data (or other non-standard gait data) are introduced, and the lateral components of the mixed gait are clearly labeled and trained, the model can be extended to monitor the lateral motion components of various mixed poses. This not only applies to the monitoring of daily irregular walking, but also provides technical support for more complex gait pattern recognition in the field of rehabilitation or motion analysis.

## Conclusion

This study introduced the “Twin Brother” model, a novel dual-task learning framework fusing CNN, LSTM, NN, and SEAM. This framework was designed for simultaneous lateral gait phase classification and continuous hip joint angle estimation using EMG signals. Compared with other models, the “Twin Brother” model demonstrated superior performance in both gait recognition and hip angle estimation. Moreover, this method has shown great potential for accurate hip joint angle prediction. These results highlight the efficacy and application potential of the proposed method over existing methods for lateral walking gait analysis and precise joint angle prediction. To our knowledge, this was the first study of lateral walking based on EMG signals, which was expected to be used for lateral resistance exercise in exoskeletons. This study provided a reference for future research on lateral walking based on EMG signals. However, the data collected in this study were all from healthy people and did not include patient data. In the future, we will collect more patient data for model construction and apply the built model to the exoskeleton system for validation research. The “Elder Brother” module in this study is capable of recognizing the user’s lateral walking gait phases, allowing the exoskeleton to adjust its movement mode in real time according to the identified gait phases. During training, based on the EMG signals, when the GFS and FLS gait phases are detected, the system can apply appropriate resistance to engage the abductor muscles of the user’s left and right legs. Conversely, when the NDS and WDS gait phases are recognized, no resistance is applied by the motor, permitting more natural motion without unnecessary interference. The “Younger Brother” module accurately predicts the hip joint angle, providing precise reference information for the exoskeleton’s movement control. The exoskeleton’s control system adjusts the joint motor based on the predicted angle, ensuring that the hip joint movement of the exoskeleton closely matches the trajectory predicted by the model. This real-time adaptation allows the exoskeleton to offer targeted support and resistance to the user. Moreover, the predicted range of the hip joint angle serves as a constraint for the exoskeleton’s movement, preventing it from exceeding the user’s natural range of motion, thereby reducing the risk of potential injuries.

In addition to applications in the field of exoskeletons, gait phase recognition based on EMG signals can provide important value for prosthetic users. Specifically, recognizing different gait phases enables prosthetic devices to adapt more effectively to the user’s movements. During the initial phase of walking, the prosthesis can be controlled to ensure a smooth transition and minimize the impact of the first step. In the support phase, the prosthesis can provide additional stability and support, helping to prevent falls caused by an unstable center of gravity. During the push phase, the device can adjust the motor to regulate the push force, propelling the user forward and optimizing movement efficiency. In the swing phase, the swing amplitude of the prosthesis is precisely adjusted by electric control, making the pace more natural and smooth. For the elderly population, the importance of gait and joint health is critical in preventing falls, which are a major concern. By identifying gait phases and predicting joint angles through EMG signals, real-time monitoring of the elderly’s walking conditions is made possible, enabling early detection of potential fall risks. For instance, when instability is detected during the support phase, it may indicate a loss of balance, prompting the system to issue a timely alert for intervention. Furthermore, predicted joint angle data can contribute to building a fall prediction model. If an abnormal joint angle is detected while walking, the system can issue an alarm, notifying the elderly person or their caregivers about a potential risk of falling.

## Data Availability

Data to support the findings of this study are available from the first author upon request.
